# Profiles of inpatient psychiatry referrals: a 4-year analysis in a Consultation-Liaison Psychiatry service

**DOI:** 10.1186/s12888-026-08121-x

**Published:** 2026-05-16

**Authors:** Jeff Huarcaya-Victoria, David Villarreal-Zegarra, Renato D. Alarcón-Guzmán

**Affiliations:** 1 Escuela Profesional de Medicina Humana, Universidad Privada San Juan Bautista, Filial Ica, Ica, Peru; 2 Unidad de Investigación, Departamento de Psiquiatría, Hospital Nacional Guillermo Almenara Irigoyen, EsSalud, Lima Peru; 3 IDEAS Research Group, Universidad Científica del Sur, Lima, Peru; 4 Department of Psychiatry and Psychology, Mayo Clinic School of Medicine, Rochester, MN USA; 5 Universidad Peruana Cayetano Heredia, Lima, Peru

**Keywords:** Consultation-Liaison Psychiatry, Inpatient referrals, Psychiatric diagnoses, COVID-19 impact, Depression, Peru, Mental health services

## Abstract

**Background:**

Consultation-Liaison Psychiatry (CLP) services are essential for addressing the psychiatric needs of patients with complex medical conditions in general hospitals.

**Aims:**

The study aims to characterize profiles of inpatient psychiatry referrals and describe referral patterns and evaluate temporal trends between referrals, patient demographics, and psychiatric and somatic diagnoses.

**Method:**

Data from January 2020 to December 2023 were collected from a CLP service during and after the pandemic, using psychiatric diagnoses from the International Classification of Diseases, Tenth Revision (ICD-10). Statistical analyses, including interrupted time series analysis with four periods, and including linear and segmented analyses, plus selective use of autocorrelation tests, were conducted to examine referral patterns and their associations with socio-demographic factors.

**Results:**

6,105 patients were referred to the CLP Service during the study period, which was 6.73% of all hospital admissions. Internal medicine and Pneumology exhibited consistently high referrals, while services like Rheumatology and Endocrinology had lower rates. Common somatic diagnoses included neoplasms (20.7%) and respiratory diseases (9.4%), while neurotic, stress-related, and somatoform disorders were prevalent psychiatric diagnoses (42.5%). Interrupted time series analysis revealed fluctuations in monthly care visits, with notable decreases coinciding with the onset of the COVID-19 pandemic.

**Conclusions:**

Our study elucidated the characteristics of patients receiving CLP services at a major Peruvian general hospital, revealing depression as a prevalent reason for consultation, and highlighting the dynamic nature of psychiatric care delivery, especially amidst the COVID-19 pandemic.

**Clinical trials registration numbers:**

Not applicable.

**Supplementary Information:**

The online version contains supplementary material available at 10.1186/s12888-026-08121-x.

## Background

In the global context of medical practice, Consultation-Liaison Psychiatry (CLP) has evolved as a specialized field, addressing the multiple intersections of mental and physical health [1, 2]. Originating in the early 20th century, the development of CLP was significantly influenced by the need to address psychiatric issues in medically ill patients, particularly those hospitalized with complex medical conditions [3, 4]. Over the decades, CLP has become an established component of hospital-based care, integrating psychiatric and medical practice [5, 6]. There is also evidence that some CLP services have demonstrated their efficacy in various healthcare settings, contributing to improved patient outcomes and reduced length of hospital stay when implemented early [7]. Integrating psychiatric consultation into general medical care is particularly relevant in resource-constrained settings.

In low- and middle-income countries such as Peru, CLP plays an important role in healthcare systems facing financial, workforce, and structural constraints [8, 9]. Inadequate attention to medical and psychiatric comorbidities can result in a substantial burden on individual and collective healthcare systems. CLP research in low- and middle-income settings is needed to guide service organization and clinical practice.

The development of CLP in Peru is closely linked to the work of Dr. Carlos Alberto Seguin, who established the Psychiatry Service at the former Hospital Obrero (now National Hospital Guillermo Almenara Irigoyen, HNGAI) in the mid-20th century [10]. Influenced by psychosomatic medicine traditions, this service promoted structured collaboration between psychiatry and other medical specialties [11]. During the second half of the 20th century, HNGAI became a reference center for CLP in Peru, consolidating an interdisciplinary model of care [12].

In the current Peruvian context, CLP adheres to regulations and guidelines established by the Ministry of Health (MINSA). Chapter V of Peru’s Mental Health Law, enacted on March 5, 2020, makes clear that mental health services in general hospitals should be organized in the form of interdisciplinary teams providing liaison psychiatry care. It also emphasizes that any mental health approach must be primarily grounded on the protection of and respect for human rights, and focused on recovery [13]. The teaching values and advantages of this multidisciplinary, integrated work in Peru have been duly recognized. Nevertheless, in spite of the pressing needs and the significant relevance that this area of mental health has reached and holds for general hospital patient populations, research productivity has been limited [14–17]. The lack of resources and specific research data on CLP care poses a considerable challenge, hindering the ability to document the provision of specialized and effective care. Therefore, a comprehensive understanding of patient characteristics and the care they receive is necessary to improve service organization and clinical decision-making. Despite the historical relevance of CLP in Peru, contemporary large-scale, longitudinal analyses of referral patterns remain scarce, particularly those incorporating quantitative time-series approaches.

This study aimed to examine the characteristics of a large sample of patients assessed over a four-year period (2020–2023) by a CLP service in Peru, a low- and middle-income country. By systematically describing referral patterns, diagnostic profiles, and service recommendations, this study seeks to address an important gap in the regional CLP literature. The primary objectives were to describe the sociodemographic and clinical characteristics of patients assessed by the CLP service using annual and aggregated frequency analyses, and to evaluate potential changes in referral patterns and consultation volume over time, particularly in response to external influences such as the COVID-19 pandemic. Additionally, as a secondary objective, an interrupted time series analysis was conducted to assess monthly variations in psychiatric consultations using four predefined interruption periods (start of the pandemic in March 2020, January 2021, January 2022, and January 2023). We hypothesized that the COVID-19 pandemic negatively impacted the number of patient consultations conducted by the CLP service.

## Methods

### Study design

This study employed a retrospective observational design to analyze data collected from the CLP Service of the National Hospital Guillermo Almenara Irigoyen (HNGAI), a general hospital in Lima, Peru.

### Participants

The participants were patients from the CLP Service of the HNGAI in Lima, Peru. The service provides consultation to inpatients admitted to medical or surgical services and who, concurrently, present psychiatric disorders or require a thorough psychiatric assessment, diagnosis, and eventual management intervention. The study timeframe spanned from January 2020 to December 2023. Utilizing a non-probabilistic sampling method, we included all cases admitted to the CLP service, regardless of data availability. The inclusion criteria stated participants to be of legal age (> 18 years) at the time of admission to HNGAI, and individuals with incomplete data regarding the referring service were excluded from the analysed sample. Notably, age is verified using an identity document at the time of admission to HNGAI.

### Setting

As mentioned above, the HNGAI was the study site, a highly complex hospital with 960 beds, located in Lima-Peru, one of the three largest establishments administered by the Social Security system, and also a tertiary referral center for all medical specialities, including psychiatry (http://www.essalud.gob.pe/estadistica-institucional/). It provides health care services to 1,547,840 individuals with Social Security insurance. As it attends to virtually all kinds of pathological conditions, it was classified in 2015 as a Specialized Health Institute III-2, the highest level conferred by Peru’s Ministry of Health.

The Consultation-Liaison Psychiatry (CLP) Service at HNGAI responds to psychiatric consultation requests from different medical and surgical departments [17]. All patient evaluations conducted by the CLP Service have been initially documented in the hospital’s electronic medical record system. Additionally, since September 2020, all patient evaluations conducted by the CLP team have been systematically recorded in a Google Form at the time of assessment. This electronic registry ensures standardized, real-time data collection, reducing reliance on retrospective chart review. Attending psychiatrists and psychiatry residents directly input clinical information into the form, ensuring data accuracy and consistency.

### Instruments and variables

#### Psychiatric diagnoses

Psychiatric diagnoses were determined by attending psychiatrists specializing in consultation-liaison psychiatry, based on clinical evaluation and documented according to the International Classification of Diseases, Tenth Revision (ICD-10) criteria [18]. All psychiatric diagnoses identified during the assessment were recorded in the electronic medical record. However, only the primary diagnosis—the condition most relevant to the patient’s clinical presentation and treatment—was considered for analytical purposes.

#### Somatic diagnoses

The somatic diagnoses recorded in this study cover a wide range of medical conditions evaluated by attending physicians in the medical or surgical wards. They include but are not limited to cardiovascular diseases, respiratory conditions, gastrointestinal disorders, endocrine abnormalities, neurological entities, and infectious diseases.

#### Sociodemographic covariates

Several socio-demographic variables were recorded: Age (subgroups of 18–34, 35–49, 50–64, and 65 years or older), gender (men and women), marital status (single, common law or co-habitant, married, widowed or separated), level of education (none, primary, high school, technical and university levels), current employment status (not working, working or retired), living condition (alone or with others), reason for consultation, service that requested it, recommendations of the liaison psychiatry team, and prescribed medications (antipsychotics, antidepressants, mood stabilizers, anxiolytics, etc.).

### Statistical analysis

#### Sociodemographic characteristics

An analysis of frequencies and percentages of sociodemographic and clinical characteristics of participants by year of assessment and pooled analysis was performed.

#### Referral rates

Referral rates per specialty were calculated by dividing the number of psychiatric consultations by the total number of adult admissions (≥ 18 years) in each respective medical or surgical service and expressed per 100 admissions. Annual referral rates and cumulative referral rates for the 2020–2023 period were computed. When denominator data were unavailable for specific services, referral rates were not calculated. Admission data per specialty were obtained from hospital administrative records.

#### Interrupted time series analysis

We used an interrupted time series analysis with four periods—pandemic onset in March 2020 (lockdown), January 2021, January 2022, and January 2023—to assess the pandemic’s impact on monthly visits. We performed these comparisons to obtain annual estimates where possible. We included March 2020 as a critical period, as it marks the official onset of the COVID-19 pandemic in Peru. Linear regression models were used to assess whether the number of monthly visits changed during the different periods. We used segmented regression analysis with Newey-West standard errors to model the data [19]. Monthly time services were used. We considered results statistically significant at a 95% confidence level (*p* < 0.05). We used Cumby–Huizinga (Breusch–Godfrey) tests, implemented with the “actest” command, to assess autocorrelation [20]. Time series analyses were performed using STATA 18, and plots were generated using STATA 18 and the ggplot2 package from R Studio.

We controlled for potential biases in the time series analysis (main analysis) by adjusting for autocorrelation, as we assumed that monthly observations were not independent. Specifically, we used a log [1] model that assumes first-order autocorrelation, in which contiguous measurements are correlated. Furthermore, we used annual estimates (with the exception of 2020) to reduce potential bias due to seasonality.

#### Ethics

The Hospital Nacional Guillermo Almenara Irigoyen’s Institutional Review Board approved the protocol of this study (Carta N°122 CIEI-OIyD-GRPA-Essalud-2025). Throughout the process, the researchers had no access to identifying information about the participants. All participants were registered users of the hospital’s Liaison Psychiatry Service and received psychological or psychiatric care as needed. Participants did not need to provide informed consent, as the data was collected retrospectively. In addition, it was ensured that all data had been completely anonymized. The authors had access to the study data in December 2024. Our study was conducted in accordance with the Declaration of Helsinki.

The procedures to access the information involved first obtaining approval from the ethics committee. Subsequently, the responsible investigator performed the data extraction using EXPLOTADATOS (a service provided by the healthcare institution), where cases are automatically anonymized and an anonymized ID is assigned.

## Results

### Participants

**Fig. 1:**
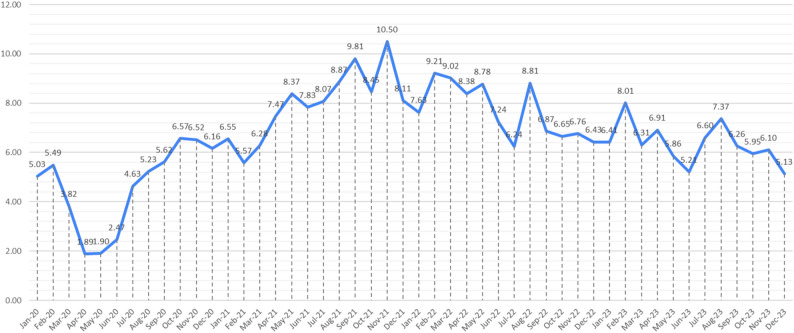
Proportion of referrals by month, between 2020 and 2023 (*n* = 6,105). Note: The denominator is the total number of referrals made in the hospital each month

During the study period, a total of 90,680 patients aged 18 years and older were admitted to the hospital. A total of 6,105 were referred to the CLP Service during the study period. On average, 127.2 consultations were performed per month (SD = 36.6), and the number of consultations per month is shown in Supplementary material 2. Figure 1 shows the proportion of referrals made to the liaison psychiatry service relative to the total number of referrals made in the hospital from January 2020 to December 2023. The rate started at 5.03% in January 2020, decreased to its lowest point at 1.90% in April 2020, and then generally increased, reaching a peak of 10.50% in November 2021. Subsequently, the rate exhibited several fluctuations, eventually declining to 5.13% by December 2023 (see Supplementary material 2).

The most frequent age group was 65 years or older (*n* = 2,448; 40.1%), and 57.4% of the sample were female (*n* = 3,502). Detailed sociodemographic characteristics and diagnoses are provided in Supplementary material 1.

The main reasons for consultation were depression (*n* = 1,381; 24.8%), mental state evaluation, which involves a general assessment of mental health using psychometric tests and a clinical interview (*n* = 1,367; 24.5%), and anxiety (*n* = 1,039; 18.6%). The main service requesting consultations was Internal medicine (*n* = 1,639; 26.8%), the most frequent Liaison Psychiatry team recommendation was initiation of medication (*n* = 3,473; 62.3%), and the most frequently prescribed medications were anxiolytics (*n* = 2,548; 41.7%) and antidepressants (*n* = 2,176; 35.6%). Sociodemographic characteristics are shown in Table 1.

**Table 1:** Sociodemographic characteristics of participants (*n* = 6,105)

	2020		2021		2022		2023		Total	
	*n*	%	*n*	%	*n*	%	*n*	%	*n*	%
Age group										
18–34	141	14.8%	212	11.7%	188	11.0%	192	11.8%	733	12.0%
35–49	184	19.3%	355	19.5%	333	19.5%	307	18.9%	1,179	19.3%
50–64	268	28.1%	545	30.0%	483	28.2%	449	27.6%	1,745	28.6%
65 to more	362	37.9%	704	38.8%	706	41.3%	676	41.6%	2,448	40.1%
Sex										
Men	411	43.0%	820	45.2%	724	42.3%	648	39.9%	2,603	42.6%
Women	544	57.0%	996	54.8%	986	57.7%	976	60.1%	3,502	57.4%
Reason for consultation*										
Aggression/Irritability	37	8.8%	97	5.3%	57	3.3%	79	4.9%	270	4.8%
Anxiety	78	18.5%	306	16.9%	378	22.1%	277	17.1%	1,039	18.6%
Self-harm	1	0.2%	3	0.2%	2	0.1%	1	0.1%	7	0.1%
Confusion	24	5.7%	141	7.8%	133	7.8%	119	7.3%	417	7.5%
Depression	126	29.9%	452	24.9%	396	23.2%	407	25.1%	1,381	24.8%
Mental state evaluation	66	15.6%	501	27.6%	423	24.7%	377	23.2%	1,367	24.5%
Previous psychiatric history	20	4.7%	109	6.0%	89	5.2%	134	8.3%	352	6.3%
Suicidal ideation	2	0.5%	8	0.4%	9	0.5%	3	0.2%	22	0.4%
Insomnia	8	1.9%	51	2.8%	85	5.0%	98	6.0%	242	4.3%
Suicide attempt	0	0.0%	1	0.1%	4	0.2%	2	0.1%	7	0.1%
Behavioral problems	9	2.1%	27	1.5%	12	0.7%	16	1.0%	64	1.1%
Psychosis	8	1.9%	9	0.5%	22	1.3%	19	1.2%	58	1.0%
Treatment refusal	3	0.7%	5	0.3%	6	0.4%	20	1.2%	34	0.6%
Psychopharmaceutical review	33	7.8%	73	4.0%	52	3.0%	45	2.8%	203	3.6%
Somatization/Conversion	1	0.2%	9	0.5%	13	0.8%	16	1.0%	39	0.7%
Follow-up	3	0.7%	13	0.7%	23	1.3%	10	0.6%	49	0.9%
Discharge instructions	3	0.7%	11	0.6%	6	0.4%	1	0.1%	21	0.4%
Recommendations of the Liaison Psychiatry team*										
No requires attention	55	13.0%	145	8.0%	186	10.9%	263	16.2%	649	11.6%
Initiation of medication	270	64.0%	1,123	61.8%	1,033	60.4%	1,047	64.5%	3,473	62.3%
Change of medication dosage	21	5.0%	73	4.0%	44	2.6%	83	5.1%	221	4.0%
Discontinue medication	8	1.9%	25	1.4%	5	0.3%	13	0.8%	51	0.9%
Brief psychotherapy	39	9.2%	318	17.5%	347	20.3%	112	6.9%	816	14.6%
Transfer to psychiatric hospitalization	1	0.2%	2	0.1%	2	0.1%	2	0.1%	7	0.1%
Complete auxiliary exams	7	1.7%	34	1.9%	18	1.1%	7	0.4%	66	1.2%
Requires the same treatment	12	2.8%	81	4.5%	72	4.2%	87	5.4%	252	4.5%
Discharge from the liaison service	9	2.1%	15	0.8%	3	0.2%	10	0.6%	37	0.7%

Note: n=number, %=Percentage, *Variables with missing data

Table 2 presents the number, proportion, and referral rates per 100 adult admissions by service and year. While internal medicine recorded the highest absolute number of referrals each year, proportional referral intensity was particularly high in Neurology and Geriatrics when expressed per 100 admissions. Notably, internal medicine recorded the highest number of referrals each year throughout the study period, with a peak of 546 referrals in 2023, representing 33.6% of all referrals to the liaison psychiatry service for that year. In contrast, services such as endocrinology and rheumatology had a lower number of referrals.

**Table 2:** Referrals by service and year, including referral rates per 100 admissions (2020–2023; *n* = 6,105)

Referring Service	2020 (*n* = 955)			2021 (*n* = 1,816)			2022 (*n* = 1,710)			2023 (*n* = 1,624)			Total (*n* = 6,105)		
	*n*	%	Referral rate	*n*	%	Referral rate	*n*	%	Referral rate	*n*	%	Referral rate	*n*	%	Referral rate
Internal Medicine	217	22.7	6.4	419	23.1	14.4	458	26.8	11.7	546	33.6	10	1,640	26.9	10.5
Pneumology	66	6.9	7.9	224	12.3	8	179	10.5	17	96	5.9	16	565	9.3	10.7
Neurology	63	6.6	20.5	125	6.9	29.4	108	6.3	30	101	6.2	25.2	397	6.5	26.6
General Surgery	50	5.2	3.6	132	7.3	7.3	94	5.5	4.1	128	7.9	5.2	404	6.6	5.1
Geriatrics	50	5.2	14.8	80	4.4	17.7	113	6.6	30	90	5.5	23.6	333	5.5	21.5
Intensive Care Unit (ICU)	21	2.2	2.7	110	6.1	17.3	67	3.9	16	45	2.8	13.2	243	4	11.2
Gynecology	65	6.8	11.7	58	3.2	6.3	50	2.9	5.6	66	4.1	6.3	239	3.9	7
Gastroenterology	24	2.5	3.8	70	3.9	9.8	53	3.1	10.2	66	4.1	10.2	213	3.5	8.5
Traumatology	29	3	3.1	63	3.5	5.7	29	1.7	1.8	59	3.6	2.8	180	2.9	3.1
Dermatology	28	2.9	13.5	42	2.3	19.4	58	3.4	20.6	43	2.6	15.4	171	2.8	17.4
COVID Unit	58	6.1	ND	88	4.8	ND	19	1.1	ND	0	0	ND	165	2.7	ND
Cardiology	28	2.9	1.2	45	2.5	1.9	45	2.6	5.2	41	2.5	4.1	159	2.6	2.4
Neurosurgery	27	2.8	5.3	35	1.9	5.6	45	2.6	5.6	45	2.8	4.3	152	2.5	5.1
Nephrology	33	3.5	7.9	33	1.8	5.2	38	2.2	4.9	45	2.8	5.1	149	2.4	5.5
Renal Transplant	24	2.5	16.3	40	2.2	21.5	72	4.2	22.7	5	0.3	2.9	141	2.3	17.1
Oncology	27	2.8	3.1	39	2.1	3.3	40	2.3	2.9	27	1.7	2	133	2.2	2.8
Obstetrics	25	2.6	0.8	19	1	0.7	30	1.8	1.1	47	2.9	1.8	121	2	1.1
Rheumatology	23	2.4	24.5	32	1.8	7.2	31	1.8	23.7	27	1.7	12.7	113	1.9	12.8
Endocrinology	13	1.4	11	21	1.2	23.9	34	2	18.9	37	2.3	18.1	105	1.7	17.8
Thoracic Surgery	8	0.8	2.8	27	1.5	6.9	27	1.6	10	31	1.9	11.4	93	1.5	7.7
Hematology	11	1.2	8	23	1.3	14.9	19	1.1	12	14	0.9	9.4	67	1.1	11.2
Urology	15	1.6	2.9	18	1	3.4	19	1.1	2.5	12	0.7	1.3	64	1	2.3
Infectious Diseases	10	1	ND	16	0.9	ND	24	1.4	ND	10	0.6	ND	60	1	ND
Liver Transplant	15	1.6	8.8	18	1	11.3	20	1.2	11.4	2	0.1	1.6	55	0.9	8.8
Plastic Surgery	11	1.2	11.6	14	0.8	12.4	14	0.8	10.1	15	0.9	8.1	54	0.9	10.2
Head and Neck Surgery	8	0.8	3.6	7	0.4	2.4	12	0.7	3.1	14	0.9	2.8	41	0.7	2.9
Breast Pathology	3	0.3	1	11	0.6	2.3	2	0.1	0.5	1	0.1	0.2	17	0.3	1
Otorhinolaryngology	1	0.1	0.6	4	0.2	2.5	5	0.3	1.6	5	0.3	1.2	15	0.2	1.4
Ophthalmology	2	0.2	1.8	1	0.1	1.6	5	0.3	7.5	5	0.3	3.9	13	0.2	3.5
Immunology	0	0	ND	1	0.1	ND	0	0	ND	1	0.1	ND	2	0	ND
Hand Surgery	0	0	0	1	0.1	0.4	0	0	0	0	0	0	1	0	0.2

Referral rates were calculated as the number of psychiatric consultations divided by the total number of adult admissions per service, expressed per 100 admissions. ND = denominator not available

The rate of missing data varied across variables. Sociodemographic and reference variables were reported adequately, with no missing data for age group or sex. The variable’s reason for consultation and recommendations of the Liaison Psychiatry team also exhibited high response rates (*n* = 5,572; 91.3%). In contrast, secondary sociodemographic variables showed moderate reporting, including civil status (*n* = 5,045; 82.6%), level of education (*n* = 4,837; 79.2%), current employment status (*n* = 4,836; 79.2%), and cohabitation status (*n* = 4,945; 81.0%).

### Somatic and psychiatric diagnoses

The most common physical diseases among the referred cases were neoplasms (C00-D48) (*n* = 1,262; 20.7%), diseases of the respiratory system (J00-J99) (*n* = 573; 9.4%), and endocrine, nutritional, and metabolic diseases (E00-E90) (*n* = 553; 9.1%). The most common psychiatric diagnoses were neurotic, stress-related and somatoform disorders (F40-F48) (*n* = 2,596; 42.5%), affective disorders (F30-F39) (*n* = 1,267; 20.8%), and organic mental disorders including symptomatic conditions (F00-F09) (*n* = 1,251; 20.5%). The gender and year distributions for each diagnosis are shown in Supplementary material 3.

### Interrupted time series analysis

**Fig. 2:**
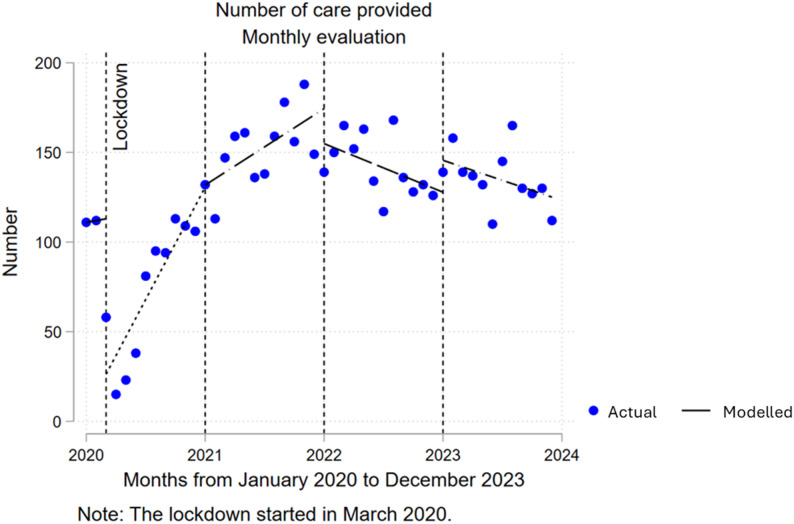
The number of cares provided between 2020 and 2023 (*n* = 6,105)

In March 2020, there was a decrease in the number of attendances compared to January and February of the same year (coeff=-86.76 [-118.72; -54.81]; *p* < 0.001), a finding that could be attributed to the initial impact of the COVID-19 pandemic. During the remainder of 2020, there was a significant trend towards an increase in the number of visits per month (coeff = 9.44 [4.52; 14.35]; *p* < 0.001). However, in 2021, although there was an increase in the number of visits, a negative trend in such number per month was found (Fig. 2), it was lower than expected when compared to previous months (coeff=-6.88 [-12.36; -1.41]; *p* = 0.015; see Table 3). Similarly, in January 2022, a decrease in the number of care visits per month was observed (coeff=-19.50 [-38.10; -0.90]; *p* = 0.040), compared to what was projected in the previous months, and during the year 2022, a decreasing monthly trend was found (coeff=-5.81 [-9.32; -2.31]; *p* = 0.002). Finally, in January 2023, there was an increase in the number of care services provided when compared to the previous months (coeff = 17.78 [1.90; 33.67]; *p* = 0.029).

**Table 3:** Interrupted time series analysis from 2020 to 2023 (*n* = 6,105)

	Coefficient	EE	*p*	95%CI
Slope between January and February 2020	1.00	0.00	0.000	[1.00; 1.00]
Change in intercept during lockdown (March 2020)	-86.76	15.78	**0.000**	[-118.72; -54.81]
Change in slope between March-December, 2020	9.44	2.43	**0.000**	[4.52; 14.35]
Change in intercept in January 2021	1.19	14.02	0.933	[-27.19; 29.57]
Change in slope between January-December 2021	-6.88	2.71	**0.015**	[-12.36; -1.41]
Change in intercept in January 2022	-19.50	9.19	**0.040**	[-38.10; -0.90]
Change in slope between January-December 2022	-5.81	1.73	**0.002**	[-9.32; -2.31]
Change in intercept in January 2023	17.78	7.85	**0.029**	[1.90; 33.67]
Change in slope between January-December 2023	0.39	1.26	0.758	[-2.16; 2.94]
Intercept	111.00	0.00	0.000	[111.00; 111.00]

Note: Intercept: The number of users served at the beginning of the study period. Slope between January and February 2020: The previous trend of the number of cares provided. Change in the intercept: Change in the number of cares provided in that month, concerning the number projected for the previous period. Change in slope: Trend expected from the number of attendances during the months assessed. Autocorrelation in lag (1) was considered

## Discussion

### Main findings

To our knowledge, this is among the first comprehensive analysis of psychiatry referral patterns to a Peruvian CLP Service over a four-year period that included the COVID-19 pandemic. By applying interrupted time series analysis, this study provides a quantitative assessment of how external disruptions influenced psychiatric service demand, a method rarely used in CLP research. In addition, the findings add data from Latin America, a region underrepresented in the international CLP literature. These results may inform service organization in comparable resource-constrained settings.

The overall referral rate of 6.73% observed in the study is notably higher than those reported in other investigations. For instance, the findings in similar studies from Europe and Asia typically range between 1 and 5% [6, 21, 22]. Comparatively, studies from other countries report even lower referrals: 1.8% in Italy [22], 2.2% in Spain [21], 0.7 to 6% in England [23], 1.19% in Iran [24], 0.01 to 3.6% in India [6], 0.02 to 3.6% in China [25], and 3.3% in Australia [26].

The differences in healthcare systems, training, and in the integration of psychiatric services into general hospital settings might account for these variations. This discrepancy may be attributed to several factors unique to our setting at the HNGAI. The long-standing and prominent history of CLP at our institution, dating back to the late 1940s, has likely played a crucial role in sensitizing medical staff to the importance of psychiatric evaluations [14, 27]. This historical context fosters a culture where psychiatric consultation is deeply integrated into the medical care process.

Beyond referral rates, our findings highlight that depression (24.8%), mental state evaluation (24.5%), and anxiety (18.6%) were the leading reasons for consultation. This pattern suggests that liaison psychiatry in our setting is not limited to crisis intervention but also plays a proactive role in diagnostic clarification and emotional assessment in medically complex patients. The high proportion of “mental state evaluation” requests may reflect a structured institutional culture in which non-psychiatric services actively seek psychiatric input for diagnostic refinement, perioperative assessment, or treatment planning [28].

Regarding psychiatric diagnoses, neurotic, stress-related, and somatoform disorders (F40–F48) accounted for 42.5% of cases, followed by mood disorders (20.8%) and organic mental disorders (20.5%). The predominance of F40–F48 diagnoses suggests that psychological distress associated with medical illness, hospitalization, and uncertainty remains a central driver of psychiatric morbidity in general hospital settings. The substantial proportion of organic mental disorders likely reflects the complexity of the inpatient population, particularly older adults and patients with multiple comorbidities.

Importantly, 62.3% of consultations resulted in initiation of pharmacological treatment, indicating that psychiatric intervention frequently translated into active therapeutic modification rather than purely advisory input. This finding underscores the operational impact of the CLP service within the hospital and suggests a clinically significant burden of untreated or undertreated psychiatric symptoms at the time of referral.

### Sociodemographic profile

From the socio-demographic perspective, the high proportion of individuals over the age of 65 (40.1% of referrals) highlights the crucial burden of mental health issues among older adults in the CLP context. This finding aligns with studies conducted in Canada [29] and Italy [30], where similar age distributions were observed. However, age should be interpreted considering the diagnostic distribution, particularly the proportion of organic mental disorders and mood disorders, which are commonly associated with medical comorbidity and cognitive vulnerability in older populations.

Sex distribution showed variability over time, with a higher proportion of female patients overall (57.4%). There is considerable variability in the literature regarding gender predominance in CLP referrals [5, 21, 31]. In our context, the higher proportion of female referrals may partially reflect differences in help-seeking behavior, but it may also relate to the diagnostic composition of referrals, particularly anxiety and depressive disorders, which are more frequently identified in women across healthcare settings.

### Impact of COVID-19

The number of patients referred to liaison psychiatry showed significant variability between 2020 and 2023 (see Fig. 1). The sharp decline in April 2020 reflects the initial healthcare reorganization during the COVID-19 pandemic. Similar reductions have been documented internationally [32, 33].

The subsequent increase and stabilization of referrals suggest adaptation of both medical services and psychiatric teams to pandemic-related constraints.

Notably, despite the temporary reduction in overall referrals, the demand for psychiatric evaluation persisted, reinforcing the essential role of CLP services even during systemic healthcare crises. The interrupted time series analysis provides quantitative evidence of these fluctuations, supporting the interpretation that psychiatric consultation activity is sensitive to macro-level healthcare disruptions.

### Public health implications

Understanding the profiles of inpatient referrals to the CLP Service of a general hospital has significant public health implications in countries like Peru and others with similar socio-economic contexts. By elucidating the patterns of psychiatric referrals and their interaction with medical conditions, this study provides relevant insights for healthcare policymakers and practitioners in resource-constrained settings.

CLP populations allow examination of the interaction between medical illness and psychological distress in hospitalized patients [34], particularly in the context of multimorbidity and aging [35].

Second, the distribution of referrals across medical and surgical specialties reflects the perceived need for psychiatric input throughout the hospital [36].

Third, the study highlights the prevalence of certain psychiatric and somatic diagnoses, such as neurotic, stress-related, and somatoform disorders, as well as diseases of the respiratory system and neoplasms. Understanding these prevalent conditions can guide consistent healthcare resources allocation and an adequate use of available management routes, ensuring the most appropriate interventions and subsequent follow-up support.

Moreover, the analysis of referral rates per 100 admissions reveals variation across medical specialties. Although these proportional measures allow more meaningful comparisons than absolute counts, differences should still be interpreted cautiously, as they may reflect variations in patient case-mix, baseline psychiatric morbidity, and service-specific clinical characteristics rather than differences in referral behavior alone.

Furthermore, the interrupted time series analysis provides valuable insights into the impact of external factors, such as the COVID-19 pandemic, on psychiatric care provision and utilization. By identifying periods of change in the number of care visits, healthcare systems can adapt their policies and services to better respond to emerging challenges, such as the increased demand for mental health support during public health crises.

Finally, medical and public health education, training, and research activities in academic and training centers face both a challenge and an opportunity to improve didactic, instrumental, and evaluation approaches to CLP itself as well as psychiatry, medicine, and other health professions. CLP also provides a structured setting for interdisciplinary training in hospital-based mental health care [37]. Future research should move beyond descriptive approaches and examine clinical outcomes, service effectiveness, and interdisciplinary collaboration [38].

### Strengths and limitations

This study has several limitations. First, its retrospective design and reliance on routinely collected clinical data may have introduced information bias due to incomplete or inconsistently documented records. Because only patients with complete records were included, selection bias may also have occurred, potentially excluding more complex or unstable cases.

Second, as a single-center study conducted in a tertiary referral hospital, the generalizability of the findings to other healthcare settings may be limited. The use of non-probabilistic sampling further restricts representativeness.

Third, although referral counts and referral rates per 100 admissions were calculated, referral patterns may still be influenced by non-clinical factors such as institutional culture, clinician attitudes, and service accessibility, which could lead to overestimation or underestimation of psychiatric morbidity across specialties.

Fourth, although we calculated the proportion of missing data, potential mechanisms of missingness, such as Missing Completely at Random (MCAR), Missing at Random (MAR), and Missing Not at Random (MNAR), were not explored. Nevertheless, the main variables exhibited reporting rates above 91%, suggesting that our findings would be unlikely to change substantially if multiple imputations or similar strategies had been implemented.

Fifth, we did not calculate the number of participants lacking data on the referring service and therefore excluded from the study. Although this information is unavailable, the frequency of this event is likely very low, as institutional policy requires this record for record for referral. Therefore, we assume that the number of such cases is minimal or negligible.

Finally, the COVID-19 pandemic likely introduced contextual bias by altering hospital admission patterns and referral thresholds, thereby affecting temporal comparisons.

## Conclusions

This study provides a detailed characterization of consultation-liaison psychiatry activity within a large Peruvian general hospital over a four-year period. The overall referral rate of 6.73%, the predominance of neurotic, stress-related, and somatoform disorders (42.5%), and the high proportion of consultations resulting in initiation of pharmacological treatment (62.3%) highlight the clinical relevance of CLP services in this setting.

Referral intensity was particularly high in Neurology and Geriatrics when expressed per 100 admissions, and interrupted time series analysis demonstrated measurable fluctuations during the COVID-19 pandemic. These findings contribute empirical data from a Latin American context and may inform service organization and future outcome-oriented research in low- and middle-income countries.

## Supplementary Information

Below is the link to the electronic supplementary material.

Supplementary Material 1

Supplementary Material 2

Supplementary Material 3

## Data Availability

The database can be requested from the corresponding author. The data comes from an electronic medical record, so it is not possible to share it due to privacy issues.
