# Supplementary material for: Profiles of inpatient psychiatry referrals: a 4-year analysis in a Consultation-Liaison Psychiatry service
Source: BMC Psychiatry. 2026 May 16;26:527. doi: 10.1186/s12888-026-08121-x (PMC13349155; doi:10.1186/s12888-026-08121-x)
Supplement: Supplementary file 1 — Supplementary Material 1 [file 12888_2026_8121_MOESM1_ESM.docx]

**Supplement material 1.** Detailed sociodemographic characteristics.

|  | 2020 | | 2021 | | 2022 | | 2023 | | Total | |
| --- | --- | --- | --- | --- | --- | --- | --- | --- | --- | --- |
|  | n | % | n | % | n | % | n | % | n | % |
| Civil status* |  |  |  |  |  |  |  |  |  |  |
| With couples | 220 | 57.9% | 951 | 59.2% | 893 | 58.8% | 871 | 56.6% | 2,935 | 58.2% |
| Single | 92 | 24.2% | 355 | 22.1% | 301 | 19.8% | 303 | 19.7% | 1,051 | 20.8% |
| Widower or separated | 68 | 17.9% | 301 | 18.7% | 324 | 21.3% | 366 | 23.8% | 1,059 | 21.0% |
| Level Education* |  |  |  |  |  |  |  |  |  |  |
| None | 12 | 3.3% | 18 | 1.2% | 19 | 1.3% | 20 | 1.3% | 69 | 1.4% |
| Primary education | 53 | 14.6% | 195 | 12.6% | 154 | 10.7% | 174 | 11.6% | 576 | 11.9% |
| High-school | 118 | 32.6% | 608 | 39.4% | 575 | 40.0% | 551 | 36.8% | 1,852 | 38.3% |
| Technical education | 62 | 17.1% | 239 | 15.5% | 235 | 16.4% | 247 | 16.5% | 783 | 16.2% |
| University education | 117 | 32.3% | 482 | 31.3% | 453 | 31.5% | 505 | 33.7% | 1,557 | 32.2% |
| Currently working* |  |  |  |  |  |  |  |  |  |  |
| No | 103 | 27.8% | 395 | 25.6% | 417 | 29.7% | 416 | 27.4% | 1,331 | 27.5% |
| Yes | 187 | 50.4% | 805 | 52.2% | 643 | 45.8% | 736 | 48.4% | 2,371 | 49.0% |
| Retiree | 81 | 21.8% | 342 | 22.2% | 343 | 24.4% | 368 | 24.2% | 1,134 | 23.4% |
| Lives with* |  |  |  |  |  |  |  |  |  |  |
| Only | 30 | 8.1% | 126 | 8.1% | 97 | 6.5% | 94 | 6.1% | 347 | 7.0% |
| With another person | 339 | 91.9% | 1,421 | 91.9% | 1,401 | 93.5% | 1,437 | 93.9% | 4,598 | 93.0% |
| Psychiatric diagnoses** |  |  |  |  |  |  |  |  |  |  |
| F00-F09 Organic mental disorders including symptomatic disorders | 233 | 24.4% | 382 | 21.0% | 328 | 19.2% | 308 | 19.0% | 1,251 | 20.5% |
| F10-F19 Mental and behavioral disorders due to psychoactive substance use | 10 | 1.0% | 13 | 0.7% | 17 | 1.0% | 11 | 0.7% | 51 | 0.8% |
| F20-F29 Schizophrenia, schizotypal and delusional disorders | 38 | 4.0% | 39 | 2.1% | 26 | 1.5% | 31 | 1.9% | 134 | 2.2% |
| F30-F39 Mood (affective) disorders | 153 | 16.0% | 392 | 21.6% | 392 | 22.9% | 330 | 20.3% | 1,267 | 20.8% |
| F40-F48 Neurotic, stress-related and somatoform disorders | 458 | 48.0% | 759 | 41.8% | 680 | 39.8% | 699 | 43.0% | 2,596 | 42.5% |
| F50-F59 Behavioural syndromes associated with physiological disturbances and physical factors | 7 | 0.7% | 39 | 2.1% | 33 | 1.9% | 41 | 2.5% | 120 | 2.0% |
| F60-F69 Adult personality and behavioral disorders | 20 | 2.1% | 24 | 1.3% | 18 | 1.1% | 12 | 0.7% | 74 | 1.2% |
| F70-F79 Mental retardation | 5 | 0.5% | 3 | 0.2% | 4 | 0.2% | 6 | 0.4% | 18 | 0.3% |
| F80-F89 Developmental psychological disorders | 0 | 0.0% | 0 | 0.0% | 3 | 0.2% | 0 | 0.0% | 3 | 0.0% |
| F90-F98 Behavioural and emotional disorders often occur in childhood and adolescent | 0 | 0.0% | 1 | 0.1% | 2 | 0.1% | 0 | 0.0% | 3 | 0.0% |
| Z00-Z99 | 83 | 8.7% | 207 | 11.4% | 270 | 15.8% | 234 | 14.4% | 794 | 13.0% |
| Physical illnesses** |  |  |  |  |  |  |  |  |  |  |
| A00–B99 Certain infectious and parasitic diseases | 64 | 6.7% | 120 | 6.6% | 85 | 5.0% | 97 | 6.0% | 366 | 6.0% |
| C00–D48 Neoplasms | 191 | 20.0% | 364 | 20.0% | 344 | 20.1% | 363 | 22.4% | 1,262 | 20.7% |
| D50–D89 Diseases of the blood and hematopoietic organs and other disorders affect | 9 | 0.9% | 29 | 1.6% | 26 | 1.5% | 26 | 1.6% | 90 | 1.5% |
| E00–E90 Endocrine, nutritional and metabolic diseases | 78 | 8.2% | 152 | 8.4% | 165 | 9.6% | 158 | 9.7% | 553 | 9.1% |
| G00–G99 Diseases of the nervous system | 56 | 5.9% | 100 | 5.5% | 115 | 6.7% | 110 | 6.8% | 381 | 6.2% |
| H00–H59 Diseases of the eye and adnexa | 3 | 0.3% | 8 | 0.4% | 8 | 0.5% | 9 | 0.6% | 28 | 0.5% |
| H60–H95 Diseases of the ear and mastoid process | 2 | 0.2% | 3 | 0.2% | 5 | 0.3% | 3 | 0.2% | 13 | 0.2% |
| I00–I99 Diseases of the circulatory system | 86 | 9.0% | 168 | 9.3% | 138 | 8.1% | 120 | 7.4% | 512 | 8.4% |
| J00–J99 Diseases of the respiratory system | 62 | 6.5% | 227 | 12.5% | 172 | 10.1% | 112 | 6.9% | 573 | 9.4% |
| K00–K93 Diseases of the gastrointestinal tract | 44 | 4.6% | 112 | 6.2% | 144 | 8.4% | 139 | 8.6% | 439 | 7.2% |
| L00–L99 Diseases of the skin and subcutaneous tissues | 39 | 4.1% | 74 | 4.1% | 60 | 3.5% | 73 | 4.5% | 246 | 4.0% |
| M00–M99 Diseases of the musculoskeletal system and connective tissue | 39 | 4.1% | 88 | 4.8% | 100 | 5.8% | 78 | 4.8% | 305 | 5.0% |
| N00–N99 Diseases of the genitourinary system | 81 | 8.5% | 124 | 6.8% | 137 | 8.0% | 105 | 6.5% | 447 | 7.3% |
| O00–O99 Pregnancy, childbirth and puerperium | 14 | 1.5% | 9 | 0.5% | 12 | 0.7% | 23 | 1.4% | 58 | 1.0% |
| P00–P96 Certain conditions originating in the perinatal period | 0 | 0.0% | 0 | 0.0% | 1 | 0.1% | 1 | 0.1% | 2 | 0.0% |
| Q00–Q99 Congenital malformations, deformities and chromosome anomalies | 3 | 0.3% | 8 | 0.4% | 6 | 0.4% | 12 | 0.7% | 29 | 0.5% |
| R00–R99 Symptoms, signs, and abnormal clinical and laboratory findings, not else | 19 | 2.0% | 59 | 3.2% | 51 | 3.0% | 42 | 2.6% | 171 | 2.8% |
| S00–T98 Trauma, poisoning, and certain other consequences of external cause | 42 | 4.4% | 94 | 5.2% | 74 | 4.3% | 106 | 6.5% | 316 | 5.2% |
| V01–Y98 External causes of morbidity and mortality | 7 | 0.7% | 2 | 0.1% | 0 | 0.0% | 4 | 0.2% | 13 | 0.2% |
| Z00–Z99 Factors influencing health status and contact with healthcare services | 55 | 5.8% | 140 | 7.7% | 146 | 8.5% | 115 | 7.1% | 456 | 7.5% |
| U00–U99 Codes for special situations | 63 | 6.6% | 138 | 7.6% | 36 | 2.1% | 9 | 0.6% | 246 | 4.0% |
| Prescribed medication** |  |  |  |  |  |  |  |  |  |  |
| Antipsychotic | 278 | 29.1% | 555 | 30.6% | 394 | 23.0% | 413 | 25.4% | 1,640 | 26.9% |
| Antidepressant | 386 | 40.4% | 669 | 36.8% | 510 | 29.8% | 611 | 37.6% | 2,176 | 35.6% |
| Mood stabilizer | 32 | 3.4% | 51 | 2.8% | 45 | 2.6% | 58 | 3.6% | 186 | 3.0% |
| Anxiolytic | 433 | 45.3% | 764 | 42.1% | 696 | 40.7% | 655 | 40.3% | 2,548 | 41.7% |

Note: n=number, %=Percentage, *Variables with missing data, **Participants may have more than one diagnosis, so percentages may sum to more than 100%.
