# Supplementary material for: Profiles of inpatient psychiatry referrals: a 4-year analysis in a Consultation-Liaison Psychiatry service
Source: BMC Psychiatry. 2026 May 16;26:527. doi: 10.1186/s12888-026-08121-x (PMC13349155; doi:10.1186/s12888-026-08121-x)
Supplement: Supplementary file 2 — Supplementary Material 2 [file 12888_2026_8121_MOESM2_ESM.docx]

**Supplement material 2.** Number and percentage of visits per month (n=6,105).

| Timeline | Number of referrals to the liaison psychiatry service | % of referrals to the liaison psychiatry service | Number of total referrals in the hospital |
| --- | --- | --- | --- |
| Jan-20 | 111 | 5.03 | 2207 |
| Feb-20 | 112 | 5.49 | 2040 |
| Mar-20 | 58 | 3.82 | 1518 |
| Apr-20 | 15 | 1.89 | 794 |
| May-20 | 23 | 1.90 | 1211 |
| Jun-20 | 38 | 2.47 | 1538 |
| Jul-20 | 81 | 4.63 | 1749 |
| Aug-20 | 95 | 5.23 | 1816 |
| Sep-20 | 94 | 5.62 | 1673 |
| Oct-20 | 113 | 6.57 | 1720 |
| Nov-20 | 109 | 6.52 | 1672 |
| Dec-20 | 106 | 6.55 | 1618 |
| Jan-21 | 132 | 6.16 | 2143 |
| Feb-21 | 113 | 5.57 | 2029 |
| Mar-21 | 147 | 6.28 | 2341 |
| Apr-21 | 159 | 7.47 | 2129 |
| May-21 | 161 | 8.37 | 1924 |
| Jun-21 | 136 | 7.83 | 1737 |
| Jul-21 | 138 | 8.07 | 1710 |
| Aug-21 | 159 | 8.87 | 1793 |
| Sep-21 | 178 | 9.81 | 1814 |
| Oct-21 | 156 | 8.45 | 1846 |
| Nov-21 | 188 | 10.50 | 1790 |
| Dec-21 | 149 | 8.11 | 1837 |
| Jan-22 | 139 | 7.66 | 1815 |
| Feb-22 | 150 | 9.21 | 1629 |
| Mar-22 | 165 | 9.02 | 1829 |
| Apr-22 | 152 | 8.38 | 1814 |
| May-22 | 163 | 8.78 | 1856 |
| Jun-22 | 134 | 7.24 | 1851 |
| Jul-22 | 117 | 6.24 | 1875 |
| Aug-22 | 168 | 8.81 | 1907 |
| Sep-22 | 136 | 6.87 | 1980 |
| Oct-22 | 128 | 6.65 | 1925 |
| Nov-22 | 132 | 6.76 | 1953 |
| Dec-22 | 126 | 6.43 | 1960 |
| Jan-23 | 139 | 6.44 | 2158 |
| Feb-23 | 158 | 8.01 | 1973 |
| Mar-23 | 139 | 6.31 | 2203 |
| Apr-23 | 137 | 5.86 | 2338 |
| May-23 | 132 | 5.21 | 2534 |
| Jun-23 | 110 | 6.60 | 1667 |
| Jul-23 | 145 | 7.37 | 1967 |
| Aug-23 | 165 | 6.26 | 2636 |
| Sep-23 | 130 | 5.95 | 2185 |
| Oct-23 | 127 | 6.10 | 2082 |
| Nov-23 | 130 | 5.13 | 2534 |
| Dec-23 | 112 | 5.13 | 2183 |
