# Supplementary material for: Profiles of inpatient psychiatry referrals: a 4-year analysis in a Consultation-Liaison Psychiatry service
Source: BMC Psychiatry. 2026 May 16;26:527. doi: 10.1186/s12888-026-08121-x (PMC13349155; doi:10.1186/s12888-026-08121-x)
Supplement: Supplementary file 3 — Supplementary Material 3 [file 12888_2026_8121_MOESM3_ESM.docx]

**
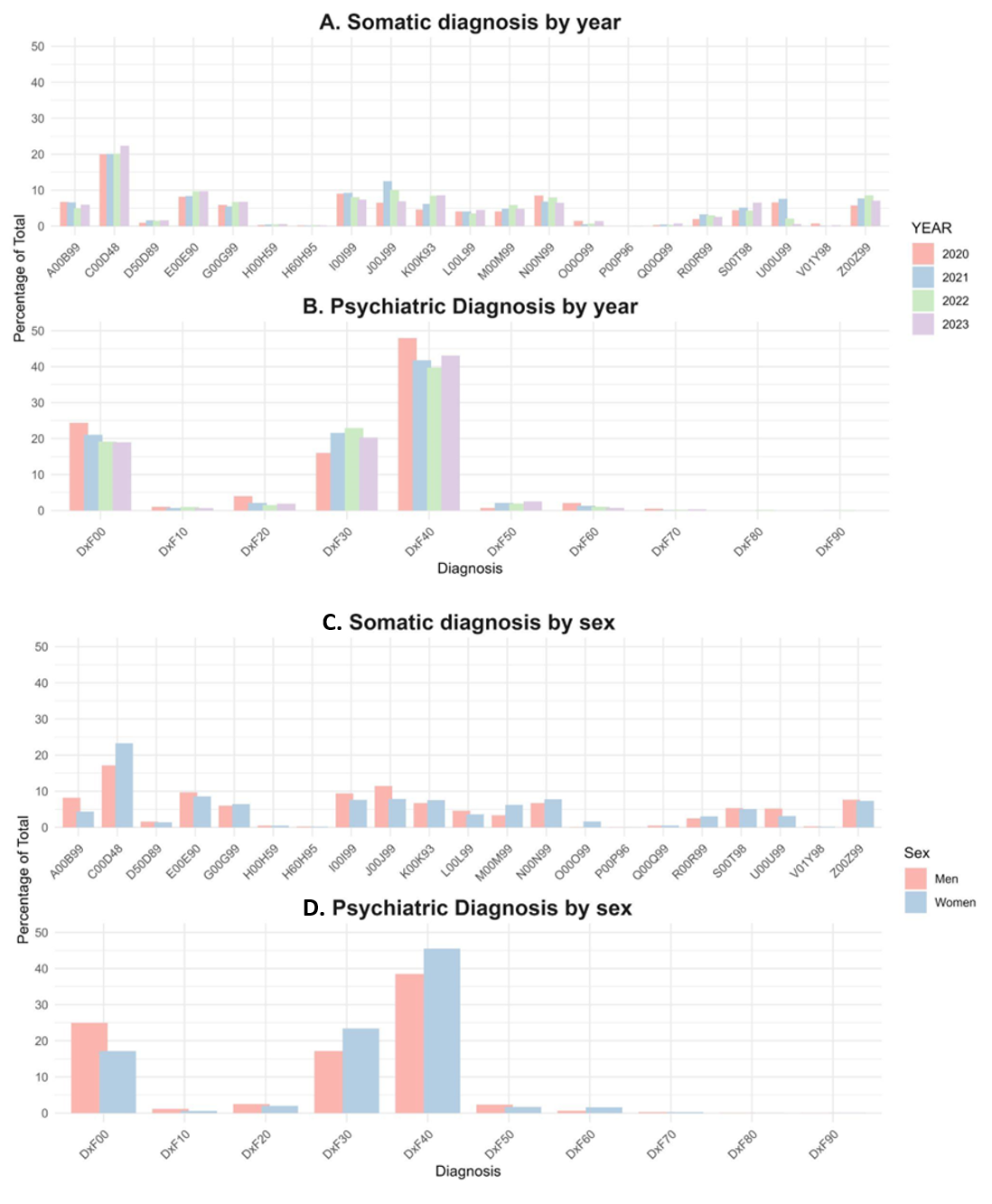
**

**Supplement material 4.** Percentage of somatic and psychiatric diagnoses: (A) and (B) and by year: (C) and (D) by sex.
